# Alterations to cavefish red blood cells provide evidence of adaptation to reduced subterranean oxygen

**DOI:** 10.1038/s41598-022-07619-0

**Published:** 2022-03-08

**Authors:** Tyler E. Boggs, Jessica S. Friedman, Joshua B. Gross

**Affiliations:** Department of Biological Sciences, University of Cincinnati, Cincinnati, OH 45221 USA

**Keywords:** Molecular biology, Evolution, Evolutionary developmental biology

## Abstract

Animals inhabiting extreme environments allow the powerful opportunity to examine adaptive evolution in response to diverse pressures. One such pressure is reduced oxygen, commonly present at high-altitude and subterranean environments. Cave-dwelling animals must also deal with darkness and starvation, both of which have been rigorously studied as key forces driving the evolution of cave-associated traits. Interestingly, hypoxia as an environmental pressure has received less attention. Here we examined putatively adaptive phenotypes evolving in a freshwater teleost fish, *Astyanax mexicanus*, which includes both surface- and cave-dwelling forms. This model system also provides the opportunity to identify convergent responses to hypoxia, owing to the presence of numerous natural and independently-colonised cave populations, alongside closely-related surface conspecifics. The focus of this study is hemoglobin, an essential molecule for oxygen transport and delivery. We found that multiple cave populations harbor a higher concentration of hemoglobin in their blood, which is coincident with an increase in cave morph erythrocyte size compared to surface fish. Interestingly, both cave and surface morphs have comparable numbers of erythrocytes per unit of blood, suggesting elevated hemoglobin is not due to overproduction of red blood cells. Alternatively, owing to an increased cell area of erythrocytes in cavefish, we reason that they contain more hemoglobin per erythrocyte. These findings support the notion that cavefish have adapted to hypoxia in caves through modulation of both hemoglobin production and erythrocyte size. This work reveals an additional adaptive feature of *Astyanax* cavefish, and demonstrates that coordinated changes between cellular architecture and molecular changes are necessary for organisms evolving under intense environmental pressure.

## Introduction

**Figure 1:**
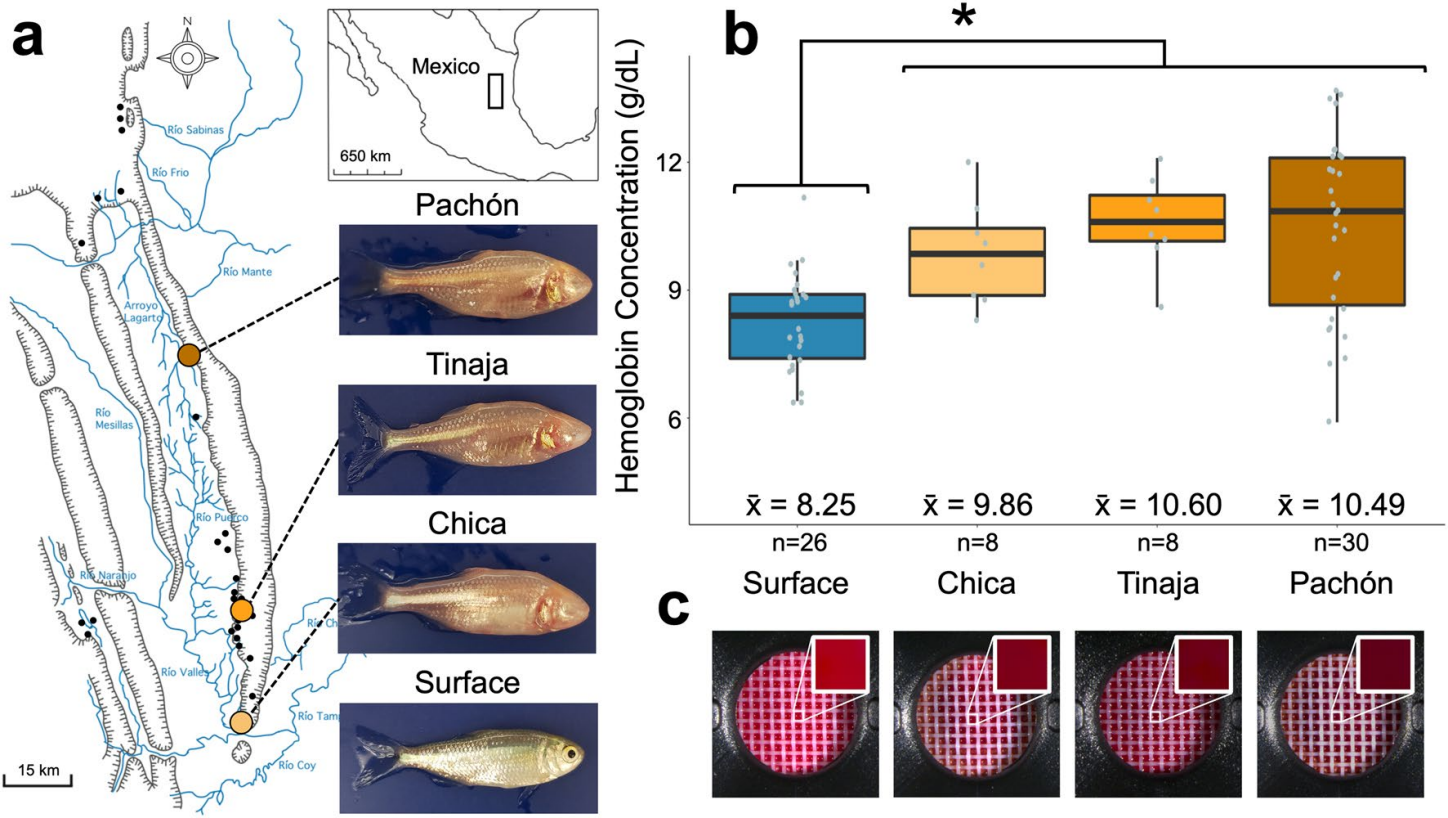
Multiple populations of *Astyanax mexicanus* cavefish have higher concentrations of hemoglobin compared to surface fish. *Astyanax mexicanus* consists of cave and surface morphotypes. The cave morphs are distributed across numerous caves of the Sierra de El Abra in northeastern Mexico, while surface morphs reside in nearby rivers, streams and lakes (**a**). Each of three cavefish populations displayed statistically significantly higher concentrations of hemoglobin than the conspecific surface morph (**b**, Chica WRS p = 0.005, Tinaja WRS p = 0.0005, Pachón WRS p = 0.0001). Hemoglobin concentrations were measured via spectrophotometric analysis (**c**). No outliers were detected.

Our understanding of the complex interactions between extreme environmental conditions and organismal response to these pressures remains incomplete. A powerful natural model to study adaptation in extreme environments is the blind Mexican cavefish, *Astyanax mexicanus* (Fig. 1a). This species allows for direct comparisons of two extant morphotypes, a river-dwelling ‘surface’ morph and obligate subterranean ‘cave’ morphs. Thirty extant cave populations^1^ are distributed across networks of subterranean caves within the exposed limestone karst of the Sierra de El Abra region of northeast Mexico (Fig. 1a). Phenotypic changes have accompanied this surface-to-cave transition, over an estimated period of ~ 20 to 500 thousand years^2,3^, including loss of eyes, pigmentation and enhancement of non-visual senses. In the 8 decades since their discovery^4^, considerable progress has been made towards understanding the mechanisms underlying regressive features, however the environmental pressures of the El Abra karst system have mostly examined the effects of limited light and nutrition^5^.

One environmental pressure commonly present in subterranean caves, reduced oxygen levels, has received far less attention. Subterranean environments across the globe frequently have reduced oxygen levels compared to surrounding terrestrial environments due to organismal respiration^6^, absence of photosynthetic input^6^, and limited air mixing with the surface atmosphere^7^. Empirical measurements of dissolved oxygen have been conducted in two El Abra caves, Pachón and Tinaja, which demonstrate significantly lower oxygen levels in cave pools compared to surrounding surface waters (DO = 2.97 mg/L in the Pachón cave compared to 8.20 mg/L in the surface environment (Rascón); 59% saturation in the Tinaja cave compared to 80% saturation in the surface environment (Nacimiento del Río Choy)^8,9^. Although this environmental feature has most likely impacted the evolution of *Astyanax* cavefish, few studies have addressed the putative adaptive features mitigating this pressure^10–12^.

Owing to the global effects of climate change^13–15^, and the consequential impact on natural dissolved oxygen levels, hypoxia tolerance has been studied in several teleost species. Complex responses to hypoxia include behavioral changes (decreased predator avoidance in grey mullets^16^), morphological changes (gill remodeling in crucian carp^17^) and molecular changes (increased expression of the oxygen sensing gene *HIF-1* in zebrafish^18^).

For sufficient oxygen to reach peripheral tissues, vertebrates utilize hemoglobin proteins as oxygen carriers. Hemoglobin molecules include four tetramers, each of which binds a single oxygen molecule, loading four oxygen molecules per hemoglobin protein. Natural oxygen concentrations can vary considerably depending on the environment, and many animals require precise oxygen levels to maintain their standard metabolic rates. Therefore, *hemoglobin* genes and proteins have likely been subject to evolutionary changes across vertebrate taxa^19^. These include numerical variation in the genomic organization of *hemoglobin* genes^20^, increasing hemoglobin concentration in tambaqui^21^, or increased hemoglobin binding affinity in red drum^22^. Here, we examined hemoglobin concentration since it is the primary molecular transport for oxygen delivery^23^.

We identified changes in cavefish impacting hemoglobin concentration and red blood cell size, which are likely adaptive in the low oxygen cave environment. These alterations, which are absent from closely-related surface morphs, likely confer hypoxia tolerance in *Astyanax* cavefish given that they were reared (and persist) under the same captive conditions for generations. Thus, this model provides the unique opportunity to identify the genetic basis for changes to hemoglobin transport and hypoxia tolerance in the wild.

## Results

### Cavefish populations harbor significantly higher blood hemoglobin concentrations compared to conspecific surface morphs

Hemoglobin is the primary oxygen transport molecule in nearly all vertebrates^19^. We first reasoned that hemoglobin levels may be higher in cave morphs as an adaptation to reduced oxygen in the cave environment (compared to overlying terrestrial environments^6^) based on direct measurements from the El Abra caves where *Astyanax* cavefish are found^8,9^. One adaptive mechanism for low oxygen is increasing hemoglobin concentration^24,25^ to augment the available oxygen for systemic transport.

Using a spectrophotometric approach (“Material and methods”), we found the mean hemoglobin concentration in surface fish (n = 26) was 8.25 ± 0.23 g/dL (Fig. 1b). All three cavefish populations examined (Chica, Tinaja and Pachón cavefish) had significantly higher mean hemoglobin concentrations than surface fish (Chica n = 8, mean = 9.86 ± 0.43 g/dL, Wilcoxon Rank-Sum (WRS) p = 0.005; Tinaja n = 8, mean = 10.60 ± 0.38 g/dL, WRS p = 0.0005; Pachón n = 30, mean = 10.49 ± 0.39 g/dL, WRS p = 0.0001; Fig. 1b), but did not differ from one another (Fig. 1b). Presumably, higher hemoglobin concentrations allow cave morphs to cope with limited dissolved oxygen in the cave. However, diverse cellular features could conceivably contribute to increased hemoglobin concentration, so we next examined cellular features of erythrocytes (i.e., red blood cells).

### Cavefish harbor a higher hematocrit compared to surface fish

**Figure 2:**
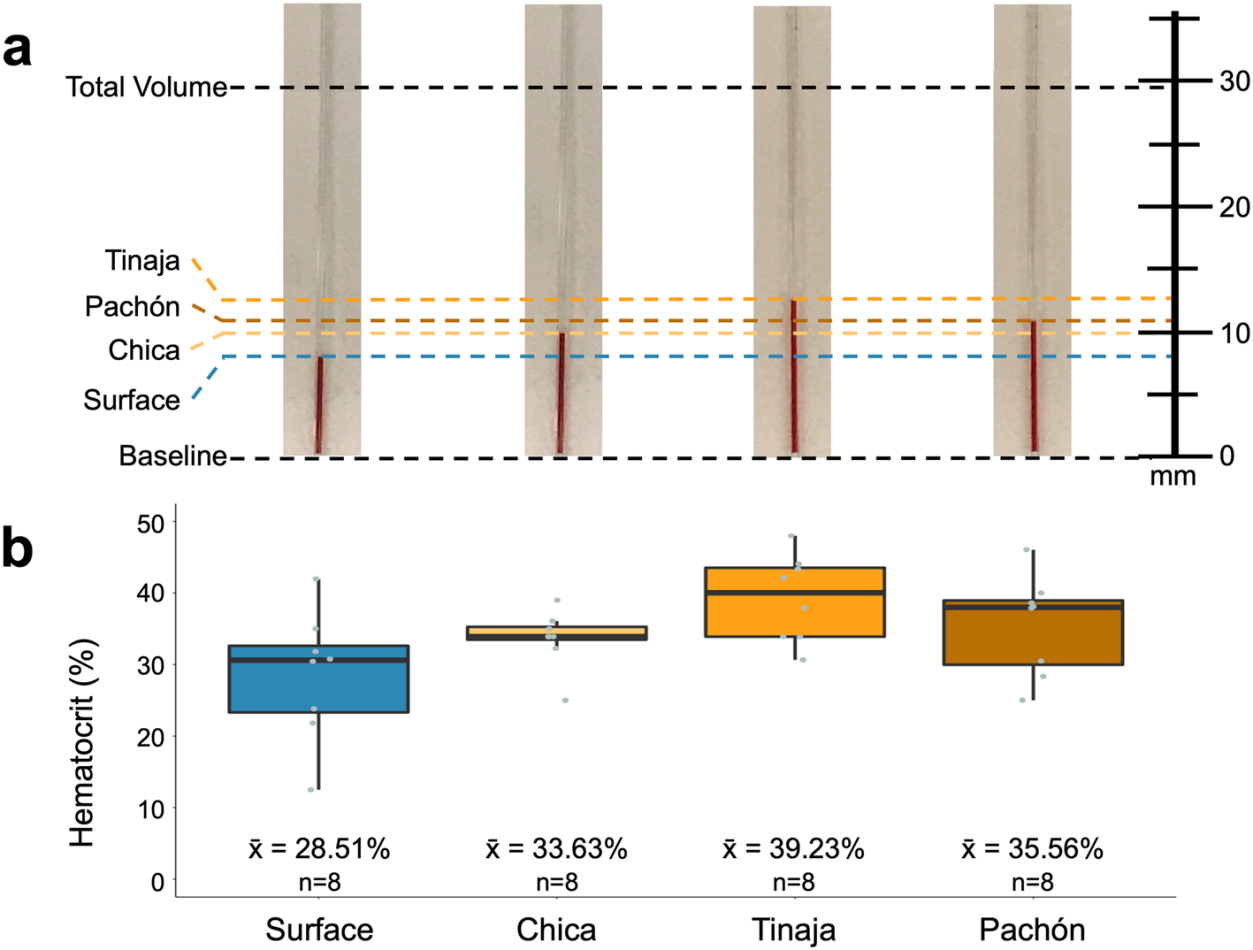
Hematocrit trends higher in cavefish than surface fish. The fraction of red blood cells to total blood volume (hematocrit) was determined via centrifugation of microcapillary tubes. The erythrocytic phase collects at the distal end of the capillary tube while the buffy coat and plasma collect more proximally (**a**). Although statistical significance was not achieved when comparing populations individually, (compared to surface; Chica WRS p = 0.115, Tinaja WRS p = 0.018, Pachón WRS p = 0.161), a clear trend of higher hematocrit was noted for each cavefish population compared to surface (**b**). When comparing by morphotype (surface against all cavefish), however, cavefish do have a statistically significant higher mean hematocrit (surface n = 8, mean = 28.51 ± 0.03%, Cave n = 24, mean = 36.14 ± 0.01%, WRS p = 0.024) than surface fish. One outlier (the lowest Chica value) was detected although statistical significance was unchanged (Table S6).

Hematocrit is the portion of a given blood volume occupied by erythrocytes (Fig. 2a). Certain clinical phenotypes in humans, e.g. polycythemia vera^26^, are diagnosed based on elevated hematocrit values^27,28^. Therefore, higher hemoglobin concentrations in cavefish may be due to presence of more erythrocytes compared to surface fish. To determine if elevated hemoglobin is mediated by overproduction of red blood cells, we compared hematocrit between surface and cave morphs.

Hematocrit measures trended higher in every cave population we examined compared to surface fish (Fig. 2b). We first performed a comparison (Wilcoxon Rank Sum test) between surface morphs and cavefish morphs (irrespective of individual cavefish localities) which revealed significant differences between these groups (surface n = 8, mean = 28.51 ± 0.03%, cave n = 24, mean = 36.14 ± 0.01%, WRS p = 0.024). We then performed a post-hoc analysis comparing each of the four populations to one another. These results clearly demonstrated the same trend, but did not achieve statistical significance following Bonferroni correction (compared to surface; Chica WRS p = 0.115, Tinaja WRS p = 0.018, Pachón WRS p = 0.161, owing to the Bonferroni correction, p values must be < 0.0083 to achieve statistical significance). The uniformly elevated hematocrit in cavefish, however, was not surprising given that cavefish have higher hemoglobin concentrations. Consistent with hemoglobin measures, the Tinaja cave population harbored the highest hematocrit value (mean hematocrit = 39.23 ± 0.03%, Fig. 2b), followed by the Pachón cave (mean hematocrit = 35.56 ± 0.03%, Fig. 2b), with the Chica cave population demonstrating the lowest hematocrit (mean hematocrit = 33.63 ± 0.01%, Fig. 2b) of the cave populations. Given these results we next examined numerical variation in red blood cells for each population.

### Erythrocyte density is comparable across *Astyanax* surface- and cave-dwelling populations

**Figure 3:**
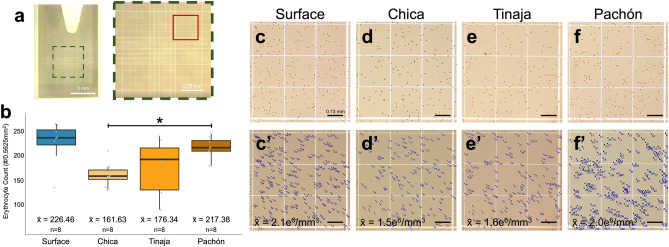
Erythrocyte density is comparable across *Astyanax* morphotypes. We determined the density of erythrocytes in a standard blood volume using a hemocytometer (**a**). Although statistical difference was detected for the Chica population (p < 0.0023), mean erythrocyte density was comparable across morphotypes (**b**). Erythrocytes were stained with Acridine Orange, visualized under light microscopy (**c**–**f**) and counted using ImageJ^29^ (v2.0.0) (**c**′–**f**′, scale bar 130 µm). Two outliers were detected (the lowest value in surface and highest in Chica). Removal of these outliers resulted in a statistically significant difference between Chica and Surface (Table S6).

We anticipated cavefish populations may have a higher density of erythrocytes than surface fish reflecting higher hemoglobin concentrations and hematocrit values. Unexpectedly, surface fish had the highest density of erythrocytes (n = 8, mean density = 2.1 × 10^6^ ± 1.4 × 10^5^/mm^3^, Fig. 3c-c′) followed closely by Pachón cavefish (n = 8, mean density = 2.0 × 10^6^ ± 8.2 × 10^5^/mm^3^, Fig. 3f-f′). Interestingly, Tinaja and Chica cavefish had slightly lower mean densities (n = 8, 1.6 × 10^6^ ± 1.8 × 10^5^/mm^3^ and n = 8, 1.5 × 10^6^ ± 6.7 × 10^4^/mm^3^, Fig. 3d–e′ respectively). Although Chica was statistically different from Pachón (WRS p = 0.001, Fig. 3b), these individuals were obtained commercially as adults (“Material and methods”) and therefore their precise age is unknown. Overall, erythrocyte densities were comparable across surface and cave morphs, which was not anticipated given the higher hemoglobin concentrations and hematocrit values in the cave populations. Since erythrocyte density was generally reduced in cavefish, we considered whether differences in erythrocyte cell size could explain the higher hemoglobin concentration in cave morphs.

### *Astyanax* cavefish harbor significantly larger erythrocytes compared to surface morphs

**Figure 4:**
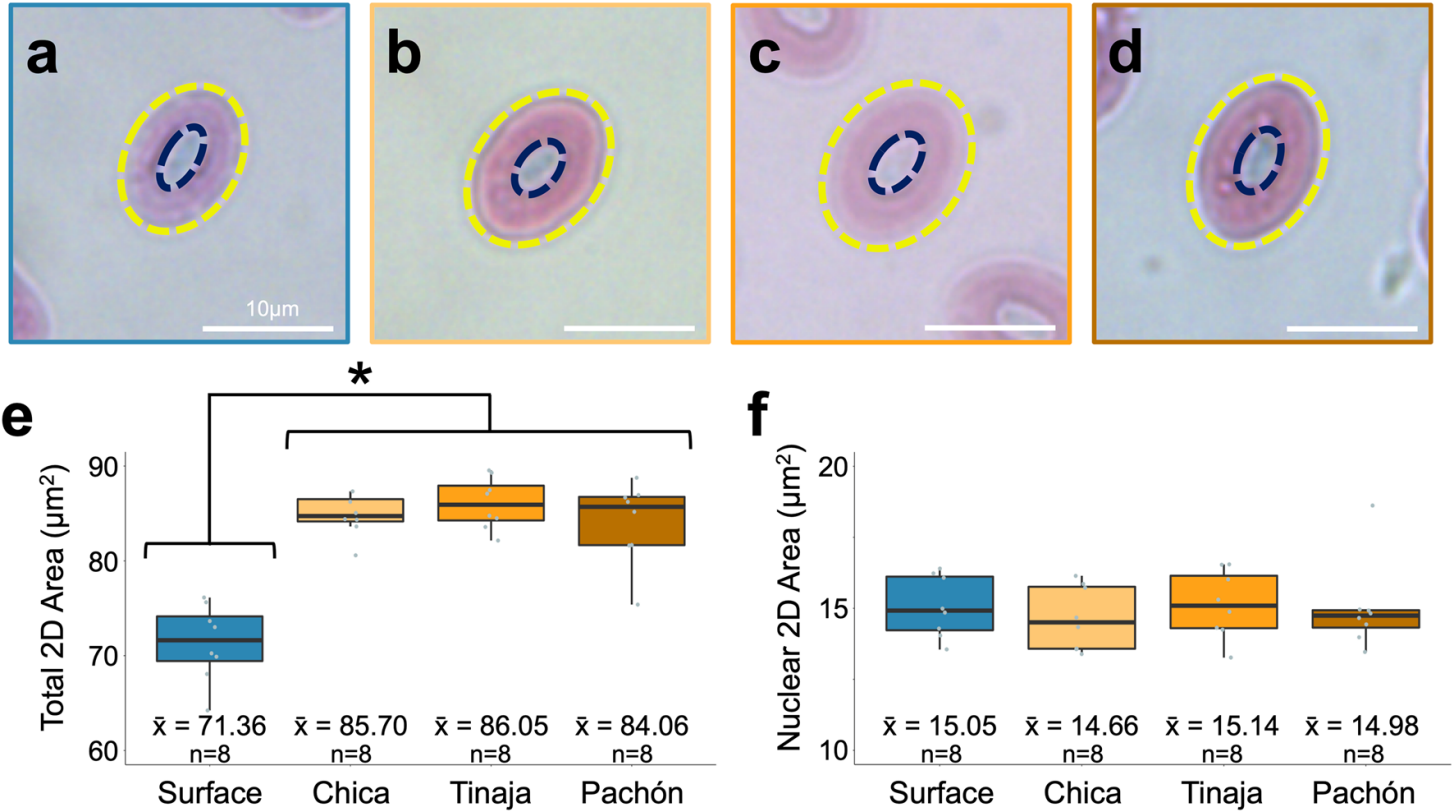
Two-dimensional surface area of cave erythrocytes is significantly larger than surface erythrocytes. Erythrocytes from each population were stained with Wright–Giemsa and visualized under light microscopy (**a**–**d**, scale bar 10 µm). The total (**a**–**d**, yellow ring) and nuclear (**a**–**d**, dark blue ring) two-dimensional area was calculated for each erythrocyte in ImageJ^29^ (v2.0.0). The total surface area was significantly higher in all cave populations compared to surface (p < 0.00003) but did not differ from each other (**e**). Although the total two-dimensional area was higher in cavefish, there were no differences in the two-dimensional area of the nuclei (**f**), suggesting the differences in total two-dimensional area are due to differences in the cytoplasm, where hemoglobin is found. Two outliers were detected (the highest Chica value and the lowest Pachón value). Removal of these outliers did not change statistical significance (Table S6).

We measured two-dimensional erythrocyte area in standard blood volumes to determine if red blood cell sizes vary across cave and surface populations. Blood smears were collected, stained and analyzed using light microscopy. Erythrocytes were unambiguously identified (“Material and methods”), manually scored in pixels units (ImageJ^29^ v2.0.0) and converted to µm for direct comparison. Since cavefish have elevated hematocrit and hemoglobin but comparable erythrocyte densities, we anticipated each cave population may show larger erythrocyte areas. Indeed, surface fish erythrocytes were the smallest across all populations (n = 8, mean area = 71.36 ± 1.43 µm^2^, Fig. 4a), while erythrocytes from the Tinaja population were on average the largest of the cave populations (n = 8, mean area = 86.05 ± 0.96 µm^2^, Fig. 4c), followed by Chica (mean area = 85.70 ± 1.38 µm^2^, Fig. 4b) and Pachón cavefish (mean area = 84.06 ± 1.52 µm^2^, Fig. 4d). Erythrocytes for all three cave populations were significantly larger than surface fish (Chica WRS p = 0.0002, Tinaja WRS p = 0.0002, Pachón WRS p = 0.0006, Fig. 4e). This cell size increase was not accompanied by an increase in nuclear area. For each population, including surface fish, the two-dimensional area of the nucleus did not differ by more than 0.5 µm^2^ (14.9 ± 0.24 µm^2^, Fig. 4f) suggesting erythrocyte area differences are attributable to increased cell cytoplasm, wherein hemoglobin is found.

### Erythrocyte size closely correlates with hematocrit values across *Astyanax* populations

**Figure 5:**
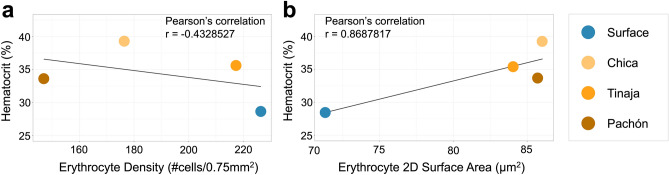
Erythrocyte size is positively correlated with hematocrit values in *Astyanax* cave and surface fish. We performed Pearson’s correlations to determine which erythrocytic feature (density or size) is correlated with hematocrit. Mean erythrocyte density was not correlated with hematocrit (r = − 0.4329, **a**). However, population erythrocyte two-dimensional surface area provided a strong, positive correlation with hematocrit value (r = 0.8688, **b**).

If differences in hematocrit between populations are not governed by increased numbers of erythrocytes, we reasoned that hematocrit variation may be correlated with differences in erythrocyte size. Qualitative comparisons seemed to demonstrate this trend, so to test this quantitatively, we performed correlations for both mean erythrocyte size and mean erythrocyte density, relative to mean hematocrit value. Erythrocyte density measures showed poor correlation to hematocrit (r = − 0.433, Fig. 5a). In contrast, erythrocyte area demonstrated a strong positive correlation with hematocrit (r = 0.869, Fig. 5b), suggesting that variation in elevated hematocrit across *Astyanax* cavefish populations is a function of erythrocyte size.

We evaluated this further by calculating mean corpuscular hemoglobin (MCH, “Material and methods”), a metric that integrates hemoglobin concentration with erythrocyte density to estimate the mass of hemoglobin in a single red blood cell. Consistent with other findings, the surface population had the lowest mean MCH value (mean MCH = 39.41 pg, Table 1) while the Chica population had the highest of the three cave populations, followed by Tinaja, and Pachón (mean MCH = 66.01 pg, 65.02 pg, and 52.20 pg, respectively; Table 1). Thus, increased erythrocyte size (linked to elevated hematocrit) likely contributes to increased hemoglobin levels in natural cavefish populations.

**Table 1:** Population-level blood parameters used to perform Pearson’s correlations and calculate mean corpuscular hemoglobin.

Population	Mean hematocrit (%)	Mean erythrocyte density (# cells*10^6^/mm^3^)	Mean erythrocyte size (µm^2^)	Mean [Hb] (g/dL)	Mean MCH (pg)
Surface	28.511	2.093	72.539	8.250	39.408
Chica	33.626	1.494	77.309	9.863	66.008
Tinaja	39.235	1.630	83.674	10.600	65.023
Pachón	35.556	2.010	81.224	10.490	52.202
	Pearson’s (r-value)	− 0.4328527	0.8687817		

*[Hb]* hemoglobin concentration, *MCH* mean corpuscular hemoglobin.

In humans, hematocrit scores provide a relative measure of the percent erythrocyte contribution to a given volume of blood, owing to the fact that red blood cell size is stable across the human population^30^. Cavefish have higher levels of hemoglobin compared to surface fish. Hematocrit levels are elevated in cavefish, but this is likely a function of larger (rather than more) erythrocytes.

## Discussion

### Adaptive phenotypes to the hypoxic cave environment in *Astyanax*

Here, we report that *Astyanax* cave morphs (from three distinct cave localities: Chica, Pachón and Tinaja) have convergently evolved higher concentrations of blood hemoglobin compared to closely-related surface fish. This elevated hemoglobin could enable cavefish to deliver more oxygen to systemic tissues per unit time. This may allow aerobic respiration to be maintained during periods of reduced oxygen to support sustained foraging activity^31–34^. Consistent with increased hemoglobin, cave morphs have higher hematocrit scores. Although this typically signals a greater density of erythrocytes^27,28^, cavefish did not have more red blood cells compared to surface fish. Rather, erythrocyte sizes varied across cave and surface populations, with cavefish uniformly harboring increased red blood cell sizes which explain, in part, their higher levels of hemoglobin.

Among the challenging environmental pressures encountered by cavefish, such as perpetual darkness and limited nutrition, include lower oxygen compared to overlying surface waters^6^. Limited oxygen has been shown to affect behavior, physiology, and certain morphologies in other teleost species^16–25,35^. Future studies in our system provide the opportunity to directly compare these features between the conspecific ‘surrogate ancestral’ morph—living in well-oxygenated environments—to derived cave morphs that have thrived in low-oxygen waters for tens to hundreds of thousands of years^2,3^.

### Larger erythrocytes as a potential mediator of higher hemoglobin levels

The discrepancy in erythrocyte size across *Astyanax* populations provides at least one explanation for increased hematocrit and hemoglobin concentration in cave morphs. Interestingly, erythrocyte size varies greatly across teleost species. Snyder and Sheafor^36^ suggested that variation in erythrocyte size maintains efficient oxygen diffusion, but as a function of varying capillary diameters. Although both cell and capillary sizes can vary across taxa, the relationship between the two remain constant. On average, erythrocyte width is 25% larger than capillary diameter^36^. Interestingly, they found no relationship between erythrocyte size and oxygen carrying capacity (including hemoglobin concentration), thus concluding that erythrocyte size varies to maintain efficient flow and diffusion.

Lay and Baldwin^37^ investigated whether nucleus size or oxygen delivery better explains erythrocyte size variation in 52 teleost species, and found an inverse relationship between erythrocyte size and hemoglobin concentration. Interestingly, they also reported an inverse relationship between erythrocyte size and aerobic swimming ability^28,38^. Specifically, fish that swim with short bursts of rapid movement tend to have smaller erythrocytes, and increased aerobic swimming was related to higher numbers of erythrocytes^39^.

One explanation for this relationship may be that a larger surface area-to-volume ratio in erythrocytes reduces oxygen diffusion distance, allowing faster loading of oxygen when needed^37^. Since volume increases faster than surface area, and cavefish erythrocytes are larger than those of the surface fish, cavefish erythrocytes likely have a lower surface area-to-volume ratio. Cave morph activity has been characterized by a higher mean velocity compared to surface fish^34^, however surface fish utilize quick, short, ‘burst’ movements^40^. Since cavefish possess fewer, but larger, erythrocytes this could reflect their differing natural activity levels. Similar results were discussed by Tang et al.^12^ in the context of differences in heart size and shape between morphotypes. Cavefish possess smaller, less triangular but therefore ‘spongier’ hearts than surface fish. Spongier hearts have a smaller ratio of compact wall tissue to trabeculae which is associated with a sedentary activity profile^41^. Further, hearts with more trabeculae have a larger surface area exposed to circulating blood, allowing for increased gas exchange which therefore may offer an advantage for cavefish in hypoxic environments^12^. 

### The underlying mechanisms governing adaptation to hypoxia

Here we measured several blood phenotypes that likely provide an adaptive advantage for *Astyanax* cavefish in hypoxic caves. Much is still unknown, however, including the underlying mechanisms that enable hypoxia resistance in this species. Future studies designed to characterize overall gene expression changes in response to hypoxia captivity^42–44^, may reveal key differentially expressed genes impacting hypoxia tolerance. For instance, certain populations of cichlids native to Lake Victoria^45,46^ demonstrate differential expression of *hemoglobin* based on variable exposure to hypoxia by ancestral populations. Additionally, coding sequence changes in *hemoglobin* genes may impact function of the encoded protein, possibly improving oxygen binding capacity in variable ambient oxygen levels over evolutionary time^20^.

A considerable body of research has identified dozens of adaptive responses to hypoxia in teleosts^47^. Other adaptations in *Astyanax* may include changes to gill anatomy and function. For instance, Moran et al.^11^ noted that Pachón cavefish gills are heavier compared to surface morphs, and the mean gill mass of Pachón/surface F_2_ hybrids was greater than the parental surface population. This may indicate positive selection occurring as a consequence of hypoxia in caves^11^. Crucian carp can ‘remodel’ their gills to improve oxygen exchange and limit unnecessary, energetically costly ion exchange^17,48^. Conceivably, similar alterations to gill morphology may provide more efficient oxygen uptake and energy conservation in hypoxic caves.

Finally, in addition to the evolution of regressive features (e.g., eye loss), many constructive non-visual sensory traits have evolved in cavefish, such as expansions of the lateral line^49,50^ and taste systems^51,52^. One unexamined sensory modality in *Astyanax* is oxygen sensing. Neuroepithelial cells (NECs) are the primary oxygen sensing cells in fish and are distributed throughout a thin epithelial layer within gill filaments and lamellae, covering the efferent aspect of the gill^53^. Owing to the positioning of these cells, they may play a role in sensing both external (ambient water) and internal (arteriole) changes in the partial pressure of oxygen^53^. Further, these cells are homologous to type I chemoreceptors located in the carotid body of mammals^54^. Key differences in ion channel expression of NECs contribute to oxygen tolerance in anoxia-resistant goldfish^55^. Given that other sensory systems are augmented as a consequence of life in the extreme cave environment, similar alterations in oxygen sensing may have similarly evolved to cope with reduced oxygen conditions.

## Material and methods

### Animal husbandry and whole blood collection

All animals used in this study were maintained in a satellite aquatic facility at the University of Cincinnati and housed in either 5- or 10-gallon tanks in a custom, reverse-osmosis husbandry unit with multiple filters (carbon, micron, UV and dense particulate filters; Aquaneering, San Diego, CA). This system conditions water to a temperature of 24 ° (± 2 °C), pH of 7.4 (± 0.2), and conductivity of 750 µS/cm (± 50 µS/cm) using real-time dose monitoring. Both *Astyanax* cave populations (Pachón, Tinaja and Chica) and surface morphs were housed under 12 h:12 h light:dark cycle and fed once daily (~ 9:00am) with a mixture of dry flake food (TetraMin Pro) and system water. Surface fish, Tinaja and Pachón cavefish used in this study were derived from breeding adult *Astyanax mexicanus* specimens originally provided by Dr. Richard Borowsky. Chica cavefish were commercially acquired, and therefore the precise age of these individuals is unknown. Certain blood parameters, including hematocrit, can change with age in humans, so we minimized biological variation in this study by excluding fish < 40 mm in standard length. Standard length measurements were not recorded for every individual, although every individual did exceed 40 mm standard length.

Animals were anesthetized using ice-cold system water and blood was collected via the caudal vein using a 31G syringe (BD Ultra-Fine™, BD Biosciences, San Jose, CA). We used fish of comparable age and standard length across all populations and found that neither factor significantly impacted any phenotypic score in this study (Supplemental Tables S1–S4). Collections occurred between 12:00 and 5:00 pm to minimize any potential diel effects. All housing conditions and collection methods were approved by the University of Cincinnati Institutional Animal Care and Use Committee (IACUC protocol # 10-01-21-01).

### Qualitative scoring

#### Hemoglobin concentration

Hemoglobin concentrations were measured with an Aimstrip Hemoglobin Meter (Germaine Laboratories, San Antonio, TX, USA). The meter was calibrated and optically verified before each use per the manufacturer’s instructions. 10 µL of whole blood was placed on a test strip (Fig. 1c) and inserted into the Hemoglobin Meter. Concentrations were automatically calculated via spectrophotometry and displayed in g/dL.

#### Hematocrit

Hematocrit values were measured via centrifugation using an LW Scientific ZipCombo centrifuge (LW Scientific, Lawrenceville, GA) outfitted with a 12-place microhematocrit rotor. Whole blood was collected into a capillary tube and sealed at the opposite end with clay. Each capillary tube was spun for 3 min at 12,000 rpm, per manufacturer’s recommendation. Blood components were separated into phases: the far more numerous and larger erythrocytes collect in the bottom phase, while the buffy coat (mostly white blood cells and platelets) and blood plasma layer on top of the erythrocytes (Fig. 2a). The length of the erythrocytic phase and the length of the total blood volume were measured. The hematocrit fraction was determined by dividing the length of the erythrocytic phase by the total blood volume.

#### Erythrocyte density

To determine the density of erythrocytes for each population, an Improved Neubauer Hemocytometer (Hausser Scientific, Horsham, PA) was used (Fig. 3a). 1 µL whole blood was diluted into a staining solution of 500 µL Phosphate Buffered Saline (1×) and 20 µL of a working solution of the nuclear stain Acridine Orange (A1301, Invitrogen by Thermo Fisher Scientific, Waltham, MA, USA). This dilution was incubated at room temperature for 20 min. Following incubation, 10 µL were placed onto the hemocytometer and visualized using a Leica DM2000 LED compound microscope (Leica Microsystems, Wetzlar, Germany). Images were captured with a Leica DMC4500 camera mounted on the microscope and Leica Application Suite (LAS) X software (version 3.0.1.15878).

The hemocytometer is equipped with intricate grid etchings (Fig. 3a) to enable a precise method of counting cell number within a precise area. The largest visible square area using our compound scope was 0.75 mm × 0.75 mm or 0.5625 mm^2^. Three different 0.5625 mm^2^ areas were counted and averaged for each individual.

To estimate the total volume, the hemocytometer was designed so that shoulders on either side of the platform support a cover slip exactly 0.1 mm above the platform. Thus, the total volume in this section is 0.05625 mm^3^. Cells counts were performed using ImageJ^29^ (v2.0.0) by first using the ‘Color Threshold’ tool. The threshold was adjusted manually for each image to eliminate optical artifacts. The square area was then selected and counting was completed using the ‘Analyze Particles’ tool. This tools permits size exclusion to further ensure artifacts are not counted. Erythrocytes are much larger than white blood cells, therefore we excluded features less than 30 µm^2^. We calculated the number of cells per 1 mm^3^ (i.e., cell density) by dividing the raw cell count by the volume (0.05625 mm^3^), and multiplying by the dilution factor (520).

#### Erythrocyte size

7.5 µL whole blood was applied to a microscope slide, smeared, air-dried and stained using Wright-Giemsa, Modified stain according to the manufacturers instruction (WG16, Millipore Sigma, Burlington, MA, USA). Nine cells were randomly selected and manually measured and averaged for each individual. The orientation of each selected cell was parallel to the slide (the short and long cell diameter at maximum length), not touching another cell, and free from obvious deformity. All cells were imaged and measured using the ‘Polygon Selection’ and ‘Measure’ tools in ImageJ^29^. Pixels were converted to µm for each image using the imaged scale bar (LAS X) during microscopy and the ‘Straight Line’ and ‘Set Scale’ tools in ImageJ^29^. The nuclear area of each erythrocyte was measured using the identical protocol employed to measure total two-dimensional area, as described above.

### Statistical analyses

 All statistical analyses were completed in R^56^ (version 3.6.1) using the base ‘stats’^56^ and ‘Rmisc’^57^ packages. At least eight individuals from each population were used for each assay as this was the greatest number of eligible individuals from the smallest in-lab population. Blinding was incorporated when possible by one investigator collecting data (imaging cells), creating an identification key, and removing individual nomenclature before a second investigator quantified and returned the data to investigator one. Wilcoxon rank-sum (WRS) tests were used to detect statistical significance for hemoglobin concentrations, hematocrit, erythrocyte density and erythrocyte size. All p values for these assays were Bonferroni corrected for multiple comparisons. Statistical significance was achieved if p < 0.0083. Pearson’s correlations were calculated in R^56^ using the base ‘stats’^56^ package. Normality was tested for each population in each assay using the Shapiro–Wilk normality test. All populations followed normal distribution with the exception of Pachón 2d nuclear area measurements (Table S5). An additional analysis was conducted to test the presence of outliers. We tested all populations in each assay using the Hampel filter^58^. This test did detect outliers, however, removing the outliers affected only a single statistical significance (Fig. 3b, Table S6.). All collected data (including outliers) is presented in this manuscript. Both male and female fish were included in all assays. Sex does not appear to be a biological factor in the blood parameters we examined, based on Wilcoxon rank-sum test analyses (n = 42 for hemoglobin concentration and n = 32 for hematocrit, erythrocyte density, and 2d area measurements, each morph included) for each assay (Supplemental Tables S1–S4). Mean corpuscular hemoglobin (MCH) estimates the mass of hemoglobin protein per erythrocyte. This value is calculated by dividing the number of grams of hemoglobin per liter by the number of red blood cells in millions per milliliter providing picograms of hemoglobin per red blood cell^59^. Scatter plots were created in R^56^ using the ‘ggpubr’^60^ package. Boxplots were created in R^56^ using the ‘ggplot2’^61^ package. In these plots, whiskers extend to the smallest or largest value not surpassing 1.5xIQR and horizontal lines represent first and third quartiles and the median. All images were edited in Microsoft PowerPoint (version 16.23).

### Ethical approval

The University of Cincinnati Institutional Animal Care and Use Committee (IACUC protocol # 10-01-21-01) approved all housing conditions and collection methods for the use of *Astyanax mexicanus* in this study. Additionally, this study conformed to the principles outlined in the Basel Declaration and the ARRIVE guidelines.

## Supplementary Information

Supplementary Information.

## Data Availability

The datasets generated during and/or analysed during the current study are available in Supplementary Information.
